# High resolution neutron Larmor diffraction using superconducting magnetic Wollaston prisms

**DOI:** 10.1038/s41598-017-00740-5

**Published:** 2017-04-13

**Authors:** Fankang Li, Hao Feng, Alexander N. Thaler, Steven R. Parnell, William A. Hamilton, Lowell Crow, Wencao Yang, Amy B. Jones, Hongyu Bai, Masaaki Matsuda, David V. Baxter, Thomas Keller, Jaime A. Fernandez-Baca, Roger Pynn

**Affiliations:** 1 Instrument and Source Division, Oak Ridge National Laboratory, Oak Ridge, TN 37831 USA; 2 Center for Exploration of Energy and Matter and Department of Physics, Indiana University, Bloomington, IN 47408 USA; 3 Quantum Condensed Matter Division, Oak Ridge National Laboratory, Oak Ridge, TN 37831 USA; 4 Department of Physics and Astronomy, The University of Tennessee, Knoxville, TN 37996 USA; 5 Faculty of Applied Sciences, Delft University of Technology, Mekelweg 15, Delft, 2629 JB The Netherlands; 6 Max-Planck-Institut für Festkörperforschung, Heisenbergstraße 1, D-70569 Stuttgart, Germany; 7 SLAC National Accelerator Laboratory, Menlo Park, CA 94025 USA

## Abstract

The neutron Larmor diffraction technique has been implemented using superconducting magnetic Wollaston prisms in both single-arm and double-arm configurations. Successful measurements of the coefficient of thermal expansion of a single-crystal copper sample demonstrates that the method works as expected. The experiment involves a new method of tuning by varying the magnetic field configurations in the device and the tuning results agree well with previous measurements. The difference between single-arm and double-arm configurations has been investigated experimentally. We conclude that this measurement benchmarks the applications of magnetic Wollaston prisms in Larmor diffraction and shows in principle that the setup can be used for inelastic phonon line-width measurements. The achievable resolution for Larmor diffraction is comparable to that using Neutron Resonance Spin Echo (NRSE) coils. The use of superconducting materials in the prisms allows high neutron polarization and transmission efficiency to be achieved.

## Introduction

The ability of conventional neutron diffraction to measure precise values of the *d*-spacings of crystalline materials is limited by factors such as the strength of the available neutron source and the practical length of neutron flight paths. The current limit is around Δ*d*/*d* of 10^−3^. At reactor neutron sources however, high resolution measurements of Δ*d*/*d* ~ 10^−6^ have been achieved using the Larmor diffraction (LD) technique^1^ first introduced by Rekveldt^2^. Like the neutron spin echo (NSE) technique proposed by Mezei^3^ for energy encoding, the LD method makes use of Larmor precession of neutron spins in well-defined magnetic fields. The method allows the lattice spacing of the diffracting crystal to be encoded into the Larmor phase of the neutron spin by making this phase depend only on the scattering vector of the diffracting Bragg peak, a quantity that is independent of the monochromaticity and collimation of the neutron beam. This enables small changes of the lattice spacing to be measured through the change of the neutron Larmor phase instead of by measuring the change in the diffraction angle.

The original Rekveldt proposal for LD involved magnetic fields before and after the sample. When the field boundaries of these two magnetic fields are aligned parallel to the crystal diffraction plane, all the diffracted neutrons will yield the same Larmor phase regardless their incident angle on the sample. Therefore, the Larmor phase of the diffracted neutrons will only depend on the geometry and intensities of the magnetic fields before and after the sample.

The LD method has been used in a number of experiments and its recent applications have been summarized by Rekveldt^4^ including absolute lattice spacing determination^5^ and temperature induced lattice variations^6^. Up to now, LD has been implemented and routinely operated on the beamlines of TRISP (FRM II, MLZ)^1^, FLEXX (BER-II, HZB)^7^ and ZETA (ILL)^8^, with a relative resolution of Δ*d*/*d* ~10^−6^. Instead of using two static magnetic fields, these instruments use four, short radio-frequency (RF) neutron spin flippers^9^ constructed using aluminium wires to minimize parasitic scattering. By physical tilting, the magnetic field boundaries of the RF flippers can be tuned to be parallel to the diffracting crystal plane of the sample. For a crystal plane with a small diffraction angle, the RF flippers have to be tilted to a large angle, forcing the neutron beam to pass through regions of the flipper where achieving field homogeneity is difficult and introducing aberrations that cause the amplitude of the Larmor oscillations to be reduced. Currently, based on our best knowledge, the maximum achievable tilting angle is 70°^10^. As the tilting angle increases, the path lengths of the neutron beam through the aluminium wires also increases, which in turn increases parasitic scattering and reduces the useable neutron flux.

As part of a project to develop neutron spin manipulation devices^11–15^, we have built a Wollaston prism that consists of two triangular shaped regions of opposing magnetic field separated by high-temperature superconducting films. As described by Li *et al*.^16–18^, using four such prisms^11^, we proposed a new technique for measuring the linewidths of dispersive phonons, which traditionally also requires tilting of the magnetic field boundaries in a similar way to the LD method described above. However, instead of physical tilting of the field boundaries, we proposed that the tuning process can be achieved by varying the magnetic field configuration in the Wollaston prisms. With this electromagnetic tuning, no physical movement of the coils is necessary and a large effective tilting angle can be achieved (up to 85°), higher than currently available. Also, since the regions of homogeneous magnetic field are defined by sharp boundaries provided by the Meissner effect of thin films of YBCO deposited on extremely flat, 0.5 mm-thick, single-crystal sapphire plates, both the transmission and polarization efficiency have been found to be high^11, 15^ in these devices.

We have used both single-arm and double-arm configurations for the experiments described in this paper. The single-arm LD experiment described by Rekveldt^4^ is less precise than the original two-arm method introduced above but it has the advantage that it does not require a zero-magnetic field region around the sample, allowing us to perform a straightforward test of the electromagnetic tuning. For both configurations, the results we obtain agree well with the dilatometry method^19^ and the results show that the principle of measuring phonon life time we proposed^16^ is viable.

**Figure 1:**
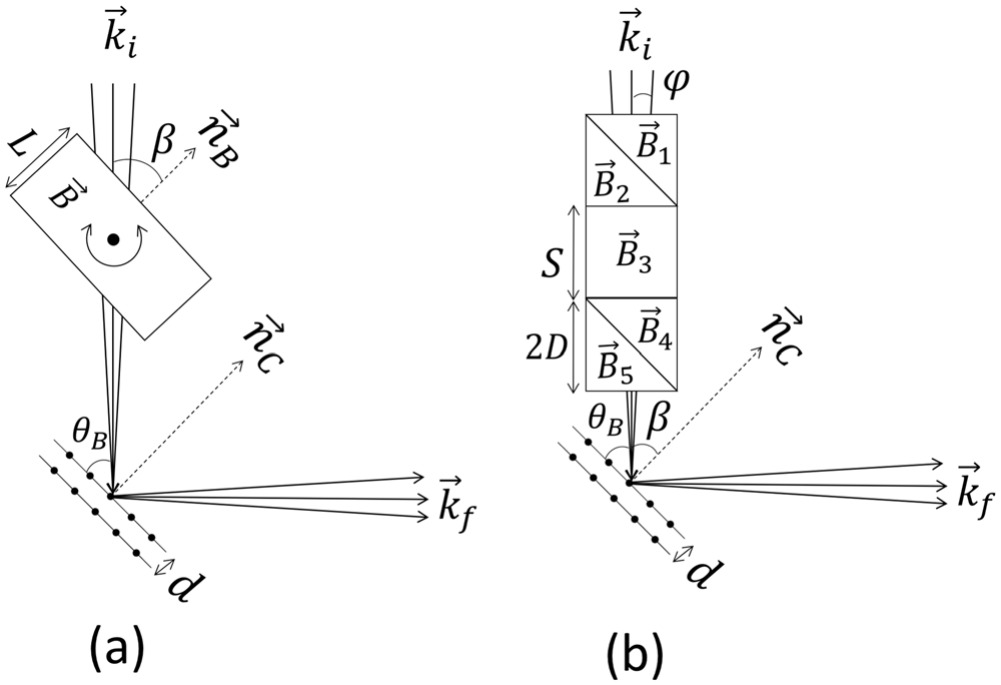
(**a**) A schematic showing the geometry of single-arm neutron Larmor diffraction, where *L* is the path length of the rectangular field perpendicular to the lattice planes and the magnetic field has intensity B. *d* is the lattice spacing. (**b**) A schematic showing the single-arm Larmor diffraction with magnetic Wollaston prisms, where the rectangular magnetic field region shown in (**a**) is replaced by two magnetic Wollaston prisms with a rectangular field region in between. S and 2D are the size of the rectangular field region and the Wollaston prisms respectively. The field intensities are B_1_, B_2_, B_3_, B_4_ and B_5_ respectively, as indicated in the schematic. For these pictures, ${\vec{n}}_{C}$ and ${\vec{n}}_{B}$ are the normals to the crystal planes and magnetic field boundary respectively.

Schematics of Larmor diffraction in the single-arm mode are shown in Fig. 1, where (a) shows the conventional setup using a single rectangular field region before the sample and (b) shows the setup we propose to use with Wollaston prisms replacing the rectangular field in (a). ${\vec{k}}_{i}$ and ${\vec{k}}_{f}$ are the incident and diffracted neutron wave vectors respectively. Though the principle of single-arm LD has been discussed in depth by Rekveldt *et al*.^4^, we briefly describe it again with modifications for our setup with Wollaston prisms.

As shown in Fig. 1(a), when neutrons pass through a rectangular field region with the polarization direction perpendicular to the magnetic field direction, neutron spins will execute Larmor precession^20^ and the accumulated Larmor phase (Φ) is given by^4^

1 $${\rm{\Phi }}=\frac{{\gamma }_{N}mBL}{h{k}_{i,\perp }}$$

where B is the field intensity inside the rectangular field region, *γ*_*N*_ and *m* are the neutron gyromagnetic ratio and mass respectively, *k*_*i*,⊥_ is the component of the incident neutron wave vector along the normal to the field boundary and *L* is the path length of the rectangular field. For a single-crystal sample placed after the rectangular field region, the required diffraction condition for the incident wave vector is given by the Bragg equation,

2 $${k}_{i,\perp }=\frac{2\pi }{d}$$

Combining Eq. 1 and Eq. 2 yields,

3 $${\rm{\Phi }}=\frac{{\gamma }_{N}mBLd}{2\pi h}$$

Equation 3 shows that, if the magnetic field boundaries are set to be parallel to the diffracting planes, all neutrons satisfying the Bragg condition in Eq. 2 will execute the same number of spin rotations as they pass through the magnetic field in Fig. 1(a). If the lattice spacing *d* is slightly changed, for example by thermal expansion, the diffracted wave vectors selected based on Eq. 2 will be slightly changed, leading to a change of Larmor phase (spin precession phase). Thus, by measuring the change in the total Larmor phase ΔΦ, the lattice distortion can be calculated as $\frac{\bigtriangleup d}{d}=\frac{\bigtriangleup {\rm{\Phi }}}{{\rm{\Phi }}}$.

Using the Larmor phase generated by the precession of the neutron spin in a rectangular field region and measuring its change due to the crystal distortion, the resolution of the setup is not strongly dependent on the collimation of the beam. This means that the resolution of the measurement can be much increased by maximizing the total Larmor phase by, for example, increasing the magnetic field and the neutron path length in the magnetic field.

As shown in Fig. 1(a), to implement Larmor diffraction, it is important to set the normal of the rectangular field boundary to be perpendicular to the lattice planes to be measured. As discussed by Li and Pynn^16^ and shown in Fig. 1(b), in order to set this field condition we propose using superconducting magnetic Wollaston prisms^11^ separated by a rectangular field region instead of the single rectangular field region shown in Fig. 1(a). By introducing the Wollaston prisms, the contributions to the total Larmor phase due to the components of the neutron wavevector parallel and perpendicular to the average wave vector can be controlled independently by changing the magnetic field configuration, as given in equation (15) in ref. 16 (refer also to Eq. 8 in the following section). For example, setting B_1_ and B_5_ to zero and all other fields equal to one another would yield a tilting angle of 45°, effectively the same as Fig. 1(a). As given by Eq. 8, the Wollaston prisms allow us to electromagnetically tune the magnetic fields to achieve an effective tilting angle analogous to the angle *β* in Fig. 1(a). By doing so, the limitation of the achievable tilting angle is relaxed to cover the full range from −90° to +90°, in principle. As discussed by Rekveldt *et al*.^2, 4^, the variation of the total Larmor phase of the single-arm configuration is sensitive to the mosaic spread of the sample to the first order, which makes it difficult to measure crystals other than perfect single-crystals. On the other hand, by introducing the second arm after the sample, the method is independent of the spread of crystal orientation to the first order, which makes it suitable for crystals with finite mosaicity. Experimental examples will be shown in the later sections.

## Results

### Tuning of the effective tilting angle using magnetic Wollaston prisms

The experiment was conducted on the HB-1 polarized triple axis spectrometer (PTAX) at the High Flux Isotope Reactor (HFIR) at Oak Ridge National Laboratory. Instead of having B_3_ and B_5_ in series, B_1_, B_3_ and B_5_ shown in Fig. 1(b) were connected in series and powered by the same power supply such that their magnetic fields were always the same including the direction and intensity (B_1_ = B_3_ = B_5_). B_2_ and B_4_ are connected in series to a separate current supply (B_2_ = B_4_). Therefore, from ref. 16, the effective tilting angle with respect to the beamline, *β*, can be calculated as

4 $$\begin{array}{rcl}\tan \,\beta  & = & -\frac{(S+2D)({B}_{4}-{B}_{5})}{2D({B}_{4}+{B}_{5})+S{B}_{5}}\\  & = & -\frac{(S+2D)(\gamma -1)}{2D(\gamma +1)+S}\end{array}$$

where S and 2D are the lengths of the central rectangular field and Wollaston prisms respectively, as shown in Fig. 1(b). Clearly, by changing the ratio γ = B_4_/B_5_, the effective tilting angle *β* can be tuned to any angle from −90° to + 90°. Note that if the sense of the hypotenuse in the Wollaston prisms is changed, the sign in Eq. 4 needs to be flipped accordingly.

Another way of understanding the effective tilting angle can be explained as follows. Based on equations (10) and (11) in ref. 16, the Larmor phase accumulated through the single diffraction arm shown in Fig. 1(b) can be written, for small divergence angles, *φ*, as

5 $${\rm{\Phi }}=\frac{{\gamma }_{N}m}{{k}_{i}}(X+Y\phi )$$

where *X* and *Y* take the values of 2D(B_1_ + B_4_) + SB_3_ and (S + 2D)(B_4_ − B_5_) respectively. Equation 5 means that all the neutrons will be encoded into a certain Larmor phase based on their wave vector and divergence. After the neutron is diffracted by the crystal plane, certain wave vectors will be selected and measured at the detector. Combining Eq. 2 and Eq. 5 yields

6 $${\rm{\Phi }}=\frac{{\gamma }_{N}md}{h}(X+Y\phi )\sin ({\theta }_{B}+\phi )$$

If we can tune the values of X and Y to eliminate the dependence of Larmor phase Φ on the divergence angle *φ* for the diffracted neutrons in Eq. (6), X and Y have to satisfy.

7 $$X+Y\phi =\frac{1}{\sin ({\theta }_{B}+\phi )}=\frac{1}{\sin \,{\theta }_{B}}(1-\phi \,\cot \,{\theta }_{B})+O({\phi }^{2})$$

Namely, the coefficients X and Y have to satisfy

8 $$\cot \,{\theta }_{B}=\,\tan \,\beta =-\frac{Y}{X}=-\frac{(S+2D)({B}_{4}-{B}_{5})}{2D({B}_{4}+{B}_{1})+S{B}_{3}}$$

which gives the same results as equation (15) in ref. 16. Thus, magnetic Wollaston prisms introduce the possibility of encoding the component of the neutron wave vector perpendicular to the average wave vector, which is currently achieved by tilting the field boundary in the conventional LD technique.

**Figure 2:**
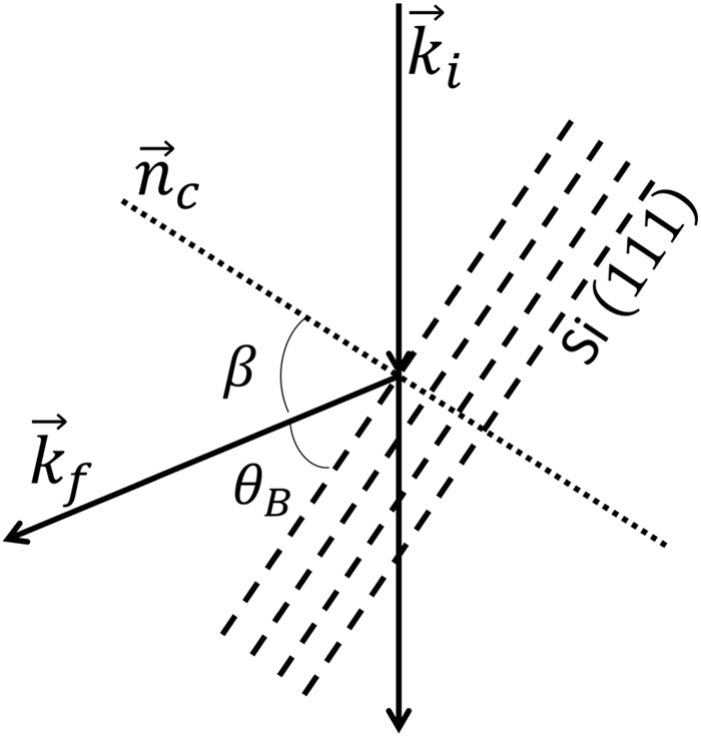
The schematic of the scattering from Si (111) planes, where 2*θ*_B_ = 46.6° at *k*_i_ = 2.55 Å^−1^ for a neutron wavelength of 2.46 Å.

The sample we used for the tuning is a perfect silicon single-crystal with a lattice parameter of *d* = 5.431 Å. The incident neutron wave vector was chosen to be 2.55 Å^−1^ (*λ* = 2.46 Å) and the scattering angle for the Si (111) plane was 2θ_B_ = 46.6°, as shown in Fig. 2. The instrument polarization efficiency for this configuration is ~85%. To set the effective tilting angle to match the crystal plane, the effective tilting angle *β* needs to be set to 66.7°, which can be obtained by tuning the ratio, γ, between B_4_ and B_5_ to obtain the highest contrast between the two polarization states of the beam (i.e. the highest flipping ratio (FR)). This ensures that all of the detected neutrons have the same Larmor phase.

**Figure 3:**
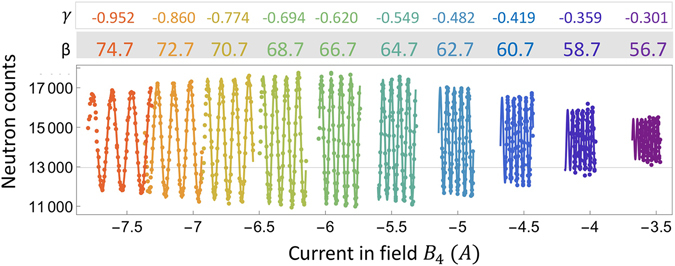
Intensities measured for the Si (111) diffraction for different ratios γ in single-arm configuration, where the fringes are obtained by scanning the current in B_4_ and B_5_ simultaneously. Different colors have different ratios between B_4_ and B_5_, and the effective tilting angles they correspond to are given on the top of the picture, along with γ, the ratio between B_4_ and B_5_. The difference between B_4_ and B_5_ is kept to be the same to avoid the effect of Larmor phase aberrations decreasing the fringe visibility. Beam size used for this tuning is 10 × 10 mm^2^.

To obtain the optimum ratio γ and thus achieve the correct effective tilting angle, we scanned B_4_ and B_5_ simultaneously while keeping their ratio, γ, constant to obtain a sinusoidal intensity fringe, as shown in Fig. 3. This fringe can be fitted to the function $I=A\cos (\omega {B}_{4})+{I}_{0}$, where A, I_0_, B_4_ and *ω* are the fringe amplitude, offset, magnetic field in B_4_ and frequency of the fringe respectively. These fringe scans were repeated for different values of γ, corresponding to different effective tilting angles. When changing the ratio between B_4_ and B_5_, the difference between them was kept the same such that the total Larmor phase accumulated in the device is the same, which means that the fringes will have the same oscillation frequency and their visibility is mainly affected by the tuning condition. The flipping ratio achieved in this tuning process is relatively low compared with the single arm measurements in the following section and this may be caused by the fact that the superconducting films inside the arm for the tuning process might have been partially damaged.

**Figure 4:**
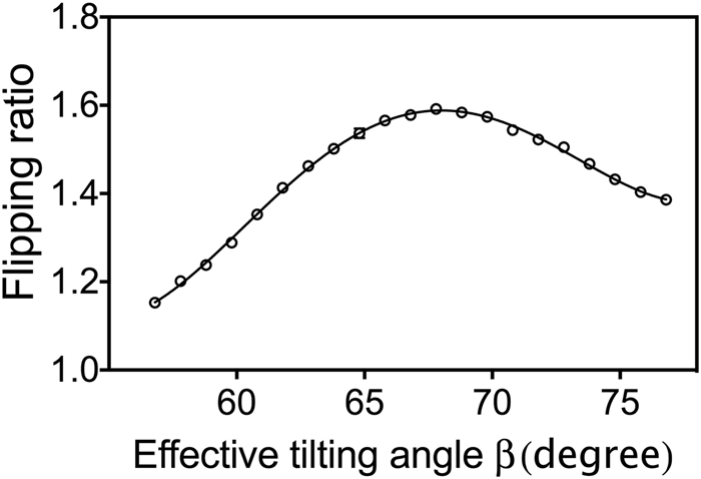
The flipping ratio of the fringes obtained at various effective tilting angles for the Si (111) diffraction plane (points) and its polynomial fit (solid curve).

The measured intensity fringes for various effective tilting angles are shown in Fig. 3. It is clear that when the field configuration is set to produce an effective tilting angle of β = 66.7°, the highest contrast fringe is obtained. The FR of the fringes in Fig. 3 is extracted and shown in Fig. 4, which is then fitted to a polynomial function. Based on the fit, the optimum effective tilting angle for the calculation of the ratio γ in Eq. 4 is 67.4° ± 0.2° instead of being 66.7°, obtained from the 2θ value. The small difference between these two values is likely due to small differences between the coils that generate the fields within the prisms. Even though the coils are wound identically, small imperfections can easily lead to small differences in the ratios between applied current and magnetic field. Thus, the experimental tuning procedure effectively verifies our contention that the tilting of the effective field boundaries can be achieved electromagnetically.

### Larmor diffraction measurements of the thermal expansion coefficient of Cu

**Figure 5:**
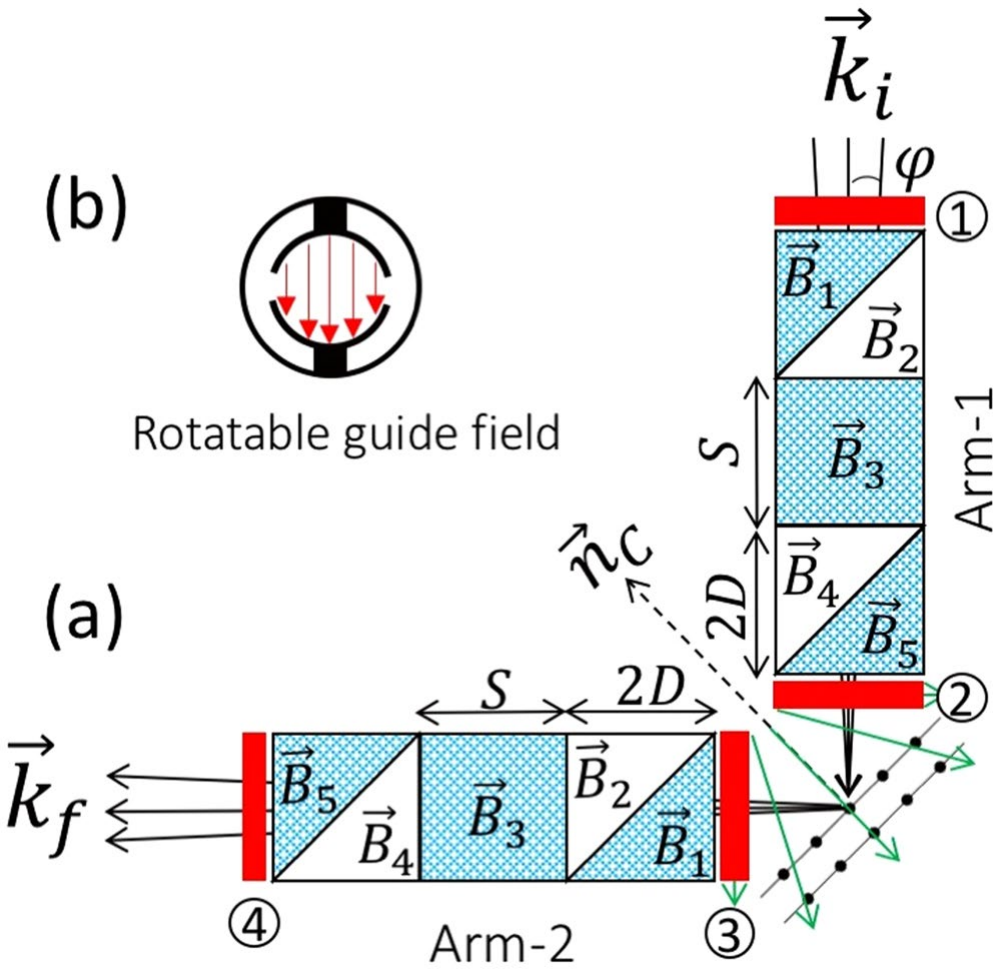
(**a**) Schematic of the double-arm Larmor diffraction. Regions with different colours refer to different magnetic field directions, where blue refers to the main field inside the device, green arrows refer to the guide field (shown in (**b**)) between the two arms and red refers to the rotatable guide field on the two ends of the device. Please note that the arm in Fig. 1(b) now goes after the sample and this causes the sense of the triangles in the arm before the sample is different from Fig. 1(b), which means the calculation of the effective tilting angle for this arm using Eq. 8 will take the opposite sign.

**Figure 6:**
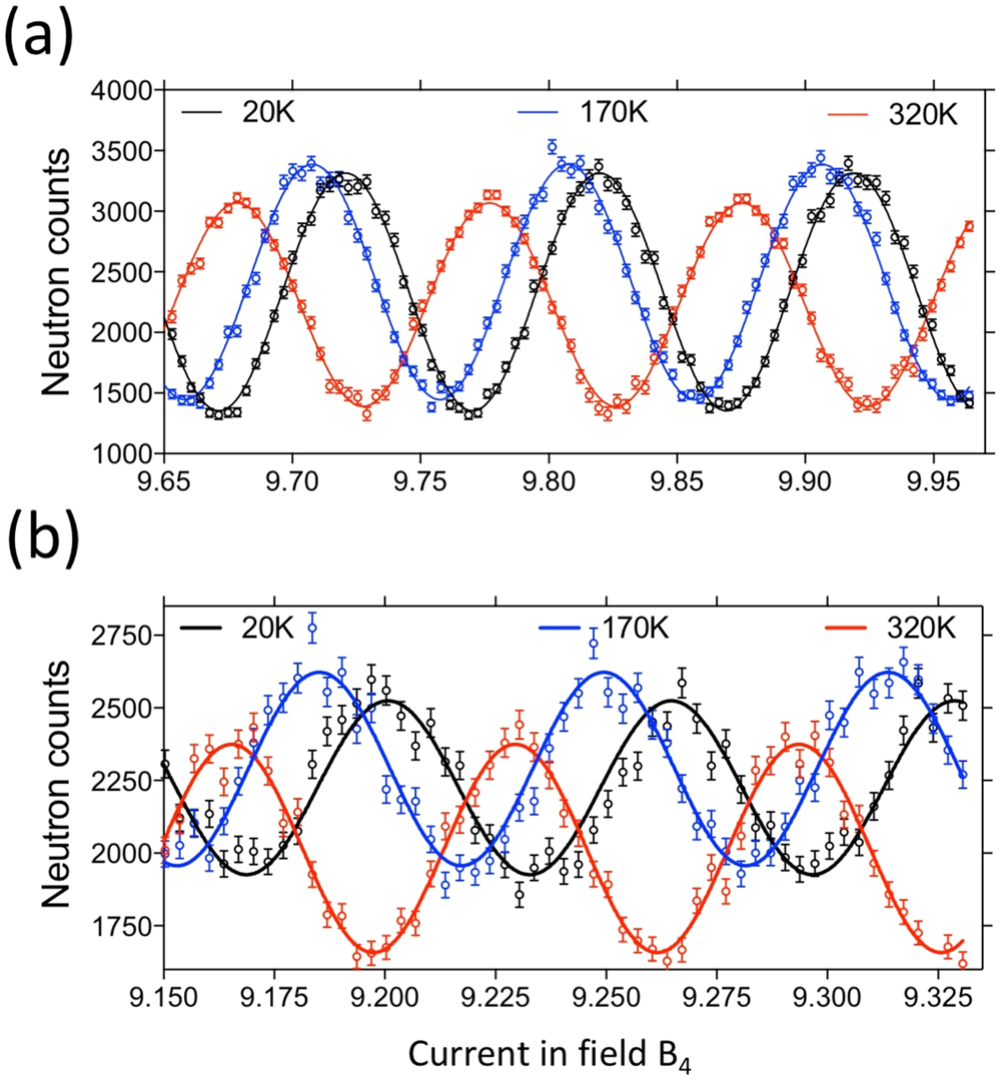
The fringes measured with single-arm (**a**) and double-arm (**b**) Larmor diffraction setup at various temperatures. Points are the measured data and solid curves are the sinusoidal fits. The change in the average neutron intensity with temperature is probably due to a slight change in the alignment of the crystal.

In the last section, the tuning procedure has been shown for a single-arm before the sample. The experimental setup we used for the actual measurement is shown in Fig. 5(a), where we have two arms with one on each side of the sample. In this schematic, different colors mean different magnetic field directions. The blue region, which is the main field inside the two arms, is always pointing in or out of the plane of the page (the vertical direction during the experiment), the green arrows in between the two arms are always in the plane. The red regions on the two ends of each arm denote rotatable guide fields. A view of these fields as seen by the neutron is shown in part (b) of Fig. 5. By simply rotating these guide fields, they can be set to any direction perpendicular to the neutron beam. For example, the first rotatable guide field can be set to be perpendicular to the field inside the device. Due to the Meissner effect produced by the superconducting film, the field regions inside and outside the prism will be well isolated yielding a non-adiabatic field transition so that the neutron spin will start to precess after entering the device. Since, when the experiment was conducted, no suitable zero-field environment was available for the sample region, two rotatable guide fields were used in between the two arms. By changing the configurations of the four rotatable guide fields, different operation modes can be achieved. For example, by setting the first and second rotatable guide fields to be perpendicular to the main field inside the first arm, while keeping the third and fourth guide field parallel to the main field, a single-arm Larmor diffraction setup could be achieved. Also, all the rotatable guide fields can be set to be perpendicular to the main field and with this configuration, only a maximum of 50% of the polarization can be achieved after the second arm, as shown later in Fig. 6(a) and (b). The setup involving a perpendicular field in between the two arms is called the ferromagnetic setup in NSE experiments, as first shown by Farago and Mezei^21^.

We measured a cylindrical single-crystal of copper at various temperatures. The diffraction plane was Cu (111) with a diffraction angle of 2θ_B_ = 72.23°, which means the effective tilting angle is β = 53.9°. The total Larmor phase at each temperature was measured by scanning the current in the device while keeping the effective tilting angle constant. Due to the temperature change, the lattice spacing will be changed and this will change the total Larmor phase. Figure 6 shows the fringes at different temperatures using both single-arm (top) and double-arm (bottom). For both configurations, the temperature induced change of the total Larmor phase can be seen through the shift of the fringes. Higher temperature will cause the crystal to expand, which will cause neutrons with larger total Larmor phase to be diffracted, based on Eq. 3. Consequently, the fringes will be shifted towards lower current. The double-arm configuration generates fringes with smaller period which means its sensitivity to a particular change in lattice spacing will be higher compared with that of the single-arm configuration. Moreover, as discussed above and also by Rekveldt *et al*.^2^, the total Larmor phase for the double-arm setup is independent of small misorientations of the diffraction planes to first order. This means, unlike the single-arm configuration, the double-arm setup is suitable for crystals with large mosaics.

**Figure 7:**
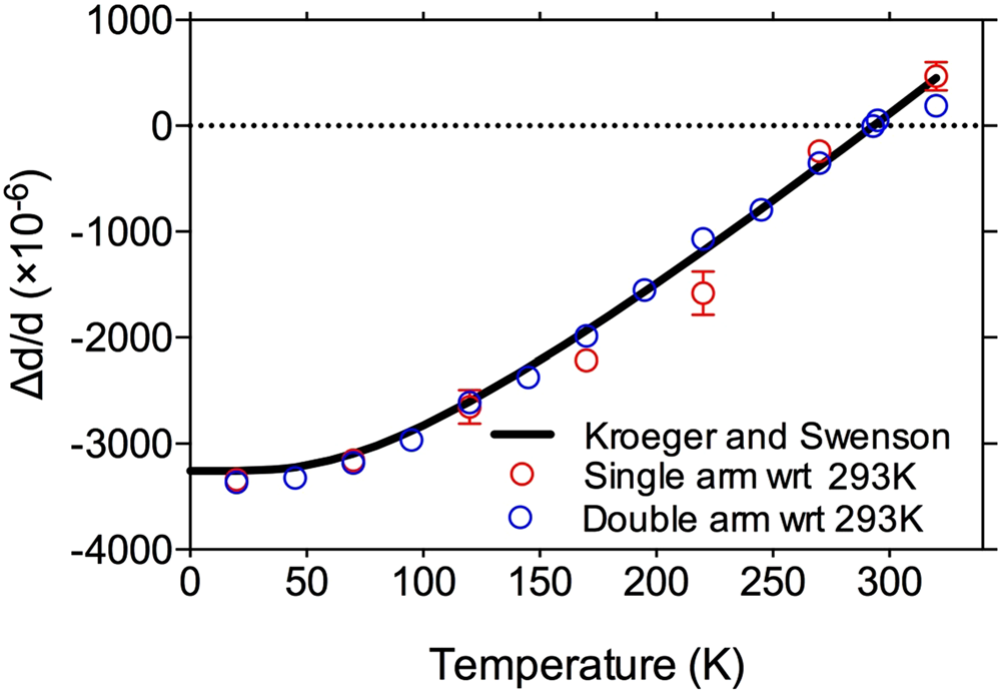
The thermal expansion values measured (points) using single-arm (red) and double-arm (blue) configurations, compared with those obtained using dilatometry^19^ (solid curve). The thermal expansion values are calculated with respect to room temperature (293.15 K).

For both single-arm and double-arm configurations, the measured thermal expansion coefficient of copper can be calculated as $\frac{\bigtriangleup d}{d}=\frac{\bigtriangleup {\rm{\Phi }}}{{\rm{\Phi }}}$, where ∆Φ is the shift of the local maximum of the fringes. The calculated thermal expansion values with respect to 293.15 K are shown in Fig. 7, where the red and blue dots are for single- and double-arm respectively. The dilatometry data^19^ is also given for comparison, shown as the solid curve in Fig. 7. We can see that the values measured by both LD configurations agree well with the dilatometry data but the double-arm data is more smoothly varying with smaller errors (the error bars for the blue points are smaller than the plotted symbol).

### Comparison between single-arm and double-arm configurations

**Figure 8:**
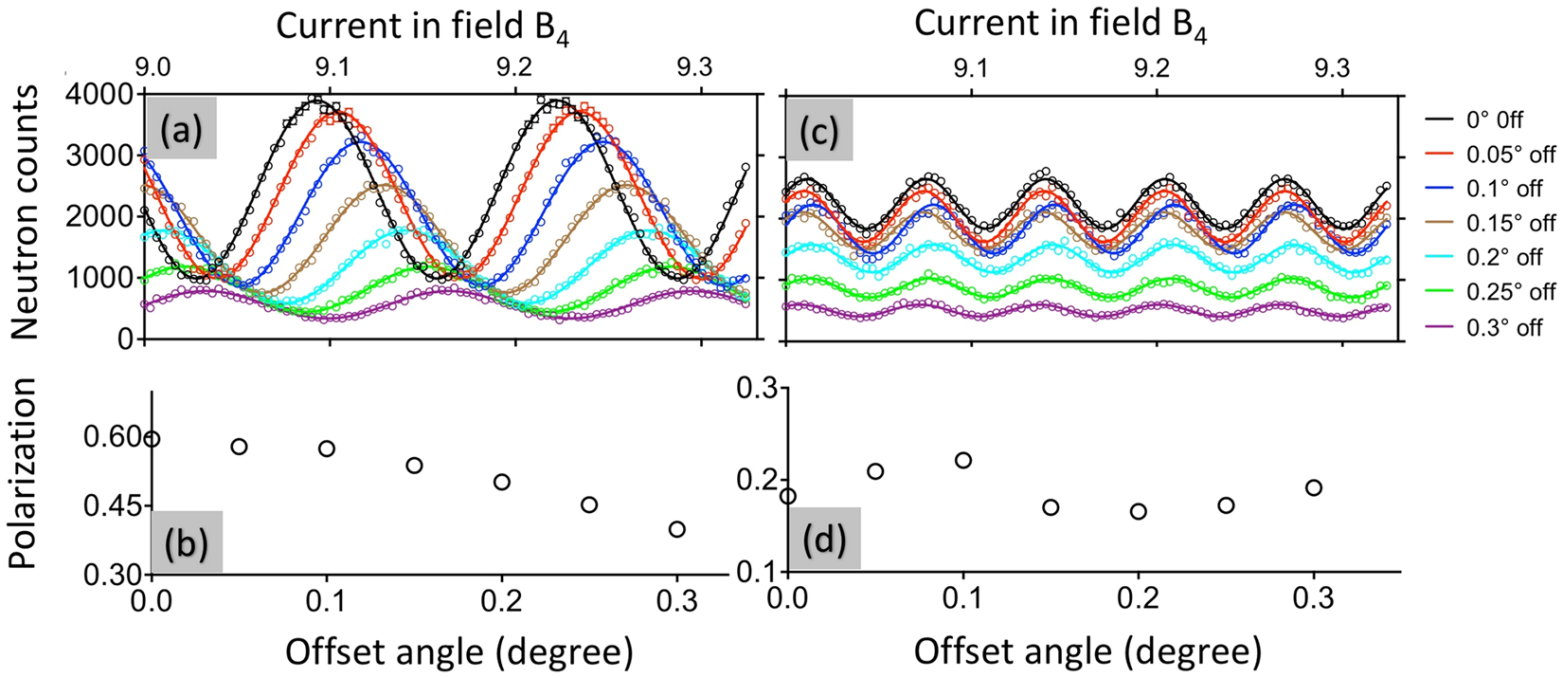
(**a**) and (**c**) are the fringes measured at various rocking angles for single-arm and double-arm configurations. The dot and solid curves are measured points and their fits respectively. (**b**) and (**d**) are the polarizations for single and double arm respectively obtained by fitting the fringes. The left side of the figure is for the single-arm configuration while the right side is for the double-arm configuration. The rocking angles are indicated on the right of the figure.

As discussed before, in addition to the higher achievable resolution, the double-arm configuration is independent of misorientation of the sample to first order. To further demonstrate this effect experimentally, we rock the sample around the vertical axis to various small angles to mimic such misorientation. For both configurations, the measured fringes are shown in Fig. 8, where the left and right figures are for the single- and double-arm methods respectively. As we can see, for both cases, the average intensity drops due to the misalignment of the sample. For single-arm, the total Larmor phase decreases monotonically as a result of the change in the wavelength selected by the crystal. The lower flipping ratio is caused by Larmor phase aberration due to the mismatch between the crystal plane and the effective tilting angle.

For the double-arm configuration, Fig. 8(c) shows that, although the average intensity drops just as it does for the single-arm, the total Larmor phase and flipping ratio stay the same for different rocking angles. These properties make it particularly useful to maintain the flipping ratio at large total Larmor phase when measuring crystals with mosaics. On the contrary, for single-arm, the fringes will be smeared out especially at large total Larmor phase. The performance of the double-arm for preserving flipping ratio will be further demonstrated in the following section.

### Balancing between the two arms of double arm configuration

**Figure 9:**
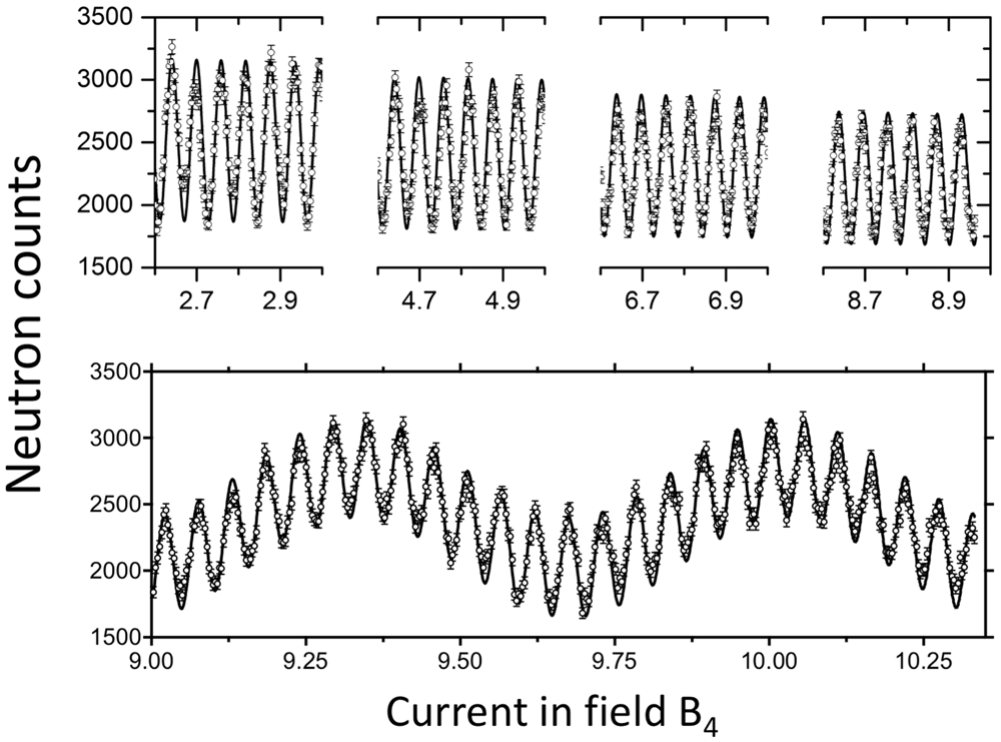
The fringes measured when the two arms are balanced (top) and unbalanced (bottom). Dots are measured data and solid curves are fits.

Since the ferromagnetic setup is used for the sample area, the measured component of the neutron polarization vector is the product of the components selected by the first and the second arm. This means the polarization efficiency measured can be simply written as $P=\,\cos \,({{\rm{\Phi }}}_{1})\cos \,({{\rm{\Phi }}}_{2})=\frac{1}{2}[\cos \,({{\rm{\Phi }}}_{1}+{{\rm{\Phi }}}_{2})+\,\cos \,({{\rm{\Phi }}}_{1}-{{\rm{\Phi }}}_{2})]$, where Φ_1_ and Φ_2_ are the total Larmor phase accumulated in the first and second arm respectively. As one can see, there is no echo point (zero Larmor phase) for this setup, as also shown by Farago and Mezei^21^. For the ferromagnetic method, it is important to have equal total Larmor phase in the two arms (on tune), otherwise a beating effect occurs, as shown in the bottom part of Fig. 9. For this plot, the effective tilting angles for the two arms are set to be 52.9° and 54.9°, which corresponds to a ratio γ of 6.67 and 8.1 between B_4_ and B_5_ respectively inside the two arms. Due to the difference between the ratios, the actual accumulated total Larmor phase inside the two arms do not balance each other, resulting in a long-period oscillation convoluted with the fringes that are used to measure the lattice expansion. As shown in the top picture of Fig. 9, when the effective tilting angle of the two arms are the same, a uniform fringe can be observed from low all the way to high Larmor phase with constant flipping ratio. These uniform fringes again show the benefit of using the double-arm configuration to minimize the Larmor phase aberration due the crystal mosaic.

## Discussion

Using newly developed superconducting magnetic Wollaston prisms, we have implemented both single-arm and double-arm Larmor diffraction, using variable currents rather than physical tilting as tuning parameters. This electrical tuning allows a large effective tilting angle can be achieved, which is particularly useful for crystal planes at small diffraction angles (2θ_B_ < 40°). The new Wollaston prisms have the added advantage that the only material in the neutron beam is sapphire plates coated with thin (300 nm) superconducting films, which have a high neutron transmission efficiency.

The operating procedure for this technique is relatively straight forward. It involves the tuning of the effective tilting angle of each arm as the first step and the subsequent scanning of the Larmor phase fringes to obtain the phase shift caused by expansion or contraction of the crystal lattice of the sample. The resolution of this technique for lattice expansion induced Larmor phase shift is given by $\frac{\bigtriangleup d}{d}=\frac{\bigtriangleup {\rm{\Phi }}}{{\rm{\Phi }}}=\frac{\bigtriangleup {\rm{I}}}{{\rm{I}}}$. For now, in our offline test, a maximum current of 25 A has been achieved without magnetically saturating the device or producing magnetic fields that penetrate the superconducting films. Considering the stability of the four power supplies used is measured to be ±0.35 mA and the step size used and current range scanned are small enough, this means that the achievable resolution of this technique with our apparatus is $\frac{\bigtriangleup d}{d}\sim 1.4\times {10}^{-5}$. Better resolution could be achieved if higher field could be achieved (we expect to double the magnetic fields in the present devices) or longer devices could be used. Unfortunately, the latter change is impractical on the HB-1 triple axis spectrometer. Considering the size of our device (0.5 m long for each arm), the achievable resolution is comparable with the resolution of the NRSE technique implemented, for example, at TRISP^1^ where a resolution of $\sim 1.5\times {10}^{-6}$ is achieved with devices that are about 0.9 m long in each arm. Beside the measurements of small crystal lattice expansion, the measurements of structural transition will be discussed and published later.
